# A public health collaboration between medical professionals and Japan's professional football league for rubella awareness

**DOI:** 10.1002/jgf2.390

**Published:** 2020-10-20

**Authors:** Toshinori Nishizawa, Yuko Murashima, Yuichi Nakamura, Keigo Sugisawa, Hironobu Nishiori, Kengo Nakamura, Noriyuki Amano, Gautam A. Deshpande, Hiroko Arioka

**Affiliations:** ^1^ Division of General Internal Medicine St. Luke's International Hospital Tokyo Japan; ^2^ Department of Ophthalmology and Visual Science Tokyo Medical and Dental University Tokyo Japan; ^3^ Department of Cardiovascular Surgery Chiba University Chiba Japan; ^4^ Department of Internal Medicine Saitama City Hospital Saitama Japan; ^5^ Amano Clinic Saitama Japan; ^6^ Department of General Medicine Juntendo University Tokyo Japan

## Abstract

A public health collaboration between medical professionals and Japan's professional football league for rubella awareness.

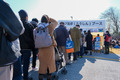

To the Editor,

Japan experienced a nationwide outbreak of rubella between 2012 and 2013, resulting in 17 000 cases of rubella and 45 cases of congenital rubella syndrome.^1^ In the latest outbreak between 2018 and 2019, 5252 cases of rubella and 5 cases of congenital rubella syndrome were reported.^2^ Most cases were adult males (78%), typically between 40 and 57 years old, who had not received routine rubella vaccination during childhood due to a cessation of the mandatory national vaccination program between 1977 and 1995. In response to the recent outbreak, the Japanese Ministry of Health, Labor and Welfare (MHLW) launched additional policies in April 2019 providing free rubella antibody testing for men in the high‐risk age cohort and, if necessary, free vaccinations for those with low antibody levels. This countermeasure is a 3‐year program aimed at increasing the population prevalence of rubella antibodies to >85% by July 2020, and eventually to increase levels to 90% by March 2022.^3^

However, as of 30 September 2019, the use of free rubella antibody testing remained quite low at less than 13%.^4^ As such, our team collaborated with Japan's professional football league (J.League) to increase rubella awareness. The average age of J.League spectators is 42.8 years old and is comprised of mainly men in their 40s (26.9%), 50s (20.5 %) and 30s (16.8 %).^5^ To access this at‐risk population, we held a free rubella antibody testing event for football spectators before an official match (FUJI XEROX SUPER CUP 2020 on 8 February 2020). In addition, prominent Japanese soccer players conducted rubella awareness activities in advance of the game through social networking platforms and print media. On the day of the event, we provided free rubella antibody tests as well as rubella awareness activities in front of the football venue (Figure 1). In total, 89 adult spectators underwent rubella antibody testing.

**Figure 1:**
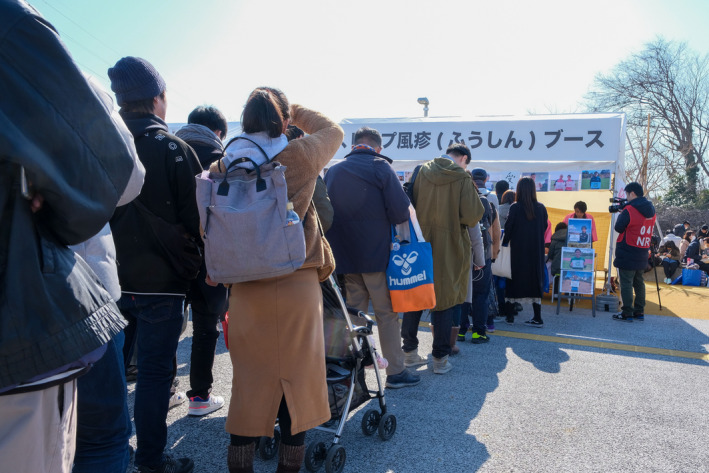
Spectators line up to be tested for rubella antibodies

We believe that antibody testing by medical professionals on the sidelines of sports events may be a useful strategy to access at‐risk populations who have little public health knowledge of rubella or ready access to antibody testing. We look forward to sharing a full report of this important public health intervention when the data become available.

## CONFLICT OF INTEREST

This event was funded by LINK‐J SCOOP 2019 and ANA Wonder FLY.

## Funding information

Author TN was supported by research funding by LINK‐J SCOOP 2019 (200 thousand yen) and ANA Wonder FLY (372 thousand yen).
